# Range dynamics, rather than convergent selection, explain the mosaic distribution of red-winged blackbird phenotypes

**DOI:** 10.1002/ece3.859

**Published:** 2013-11-07

**Authors:** Matthew J Dufort, F Keith Barker

**Affiliations:** 1 Department of Ecology, Evolution and Behavior, University of Minnesota100 Ecology Building, 1987 Upper Buford Circle, Saint Paul, Minnesota, 55108; 2 Bell Museum of Natural History, University of Minnesota100 Ecology Building, 1987 Upper Buford Circle, Saint Paul, Minnesota, 55108

**Keywords:** Demographic expansion, hybridization, microsatellite, mitochondrial DNA

## Abstract

Geographic distributions of genetic and phenotypic characters can illuminate historical evolutionary processes. In particular, mosaic distributions of phenotypically similar populations can arise from parallel evolution or from irregular patterns of dispersal and colonization by divergent forms. Two phenotypically divergent forms of the red-winged blackbird (*Agelaius phoeniceus*) show a mosaic phenotypic distribution, with a “bicolored” form occurring disjunctly in California and Mexico. We analyzed the relationships among these bicolored populations and neighboring typical populations, using ∼600 bp of mitochondrial DNA sequence data and 10 nuclear short tandem repeat loci. We find that bicolored populations, although separated by ∼3000 km, are genetically more similar to one other than they are to typical populations separated by ∼400 km. We also find evidence of ongoing gene flow among populations, including some evidence of asymmetric gene flow. We conclude that the current distribution of bicolored forms represents incomplete speciation, where recent asymmetric hybridization with typical *A. phoeniceus* is dividing the range of a formerly widespread bicolored form. This hypothesis predicts that bicolored forms may suffer extinction by hybridization. Future work will use fine-scaled geographical sampling and nuclear sequence data to test for hybrid origins of currently typical populations and to more precisely quantify the directionality of gene flow.

## Introduction

Speciation appears to take place most often in allopatry (reviewed in Coyne and Orr 2004; see also Chesser and Zink 1994; Barraclough and Vogler 2000), although other modes of speciation have a role (Berlocher 1998; Berlocher and Feder 2002; Fitzpatrick and Turelli 2006). The evolutionary fate of species formed in allopatry depends both on the evolution of isolating mechanisms and on the potential for contact with close relatives with which they might interbreed (Servedio and Noor 2003; Mallet 2005; Weir and Price 2011). Where reproductive isolation is not complete and range shifts bring nascent species into contact, hybridization is a natural consequence. However, the nature of the resulting hybridization offers unique insights into evolutionary processes, including the importance of selection in species differentiation, the role of genetic architecture in isolation, and the dynamics of species formation and loss (Endler 1977; Harrison 1993; Arnold 2006). Hybridization can have a number of outcomes, ranging from hybrid speciation (Mallet 2007), to widespread introgression and apparent fusion of the two forms (Hegde et al. 2006; DaCosta et al. 2008), to evolution of narrow tension zones with highly limited genetic exchange (Macholan et al. 2007; Mettler and Spellman 2009), possibly leading to the evolution of isolation (Servedio and Noor 2003). Introgression itself can vary not only in degree but in character, including in the symmetry of gene flow.

Asymmetry in gene flow is a particularly interesting phenomenon, as it suggests that the hybridizing forms differ in characteristics of selective value, including demographic traits (e.g., intrinsic reproductive rates, survivorship), behavioral characters (e.g., aggression, dispersal patterns), or sexual competitiveness (e.g., male territoriality or attractiveness). Such asymmetries imply that differentiation is not always a neutral process and that differences acquired in allopatry – along with the selective forces that favor them – have important implications for the success of new lineages. Moreover, these asymmetries may be an underappreciated factor in community assembly and stability (Weir and Price 2011). For instance, repeated occurrences of allopatry (e.g., driven by climatic cycles) might generate closely related forms that differ systematically due to consistent differences in their refugial ranges. If this was followed by biased extinction upon secondary contact, then the phenotypic characteristics of the species pool itself might be altered (Martin et al. 2010).

Unless counteracted by changing selective regimes, asymmetric introgression is expected to lead to extinction of the form receiving more immigrants (Rhymer and Simberloff 1996), suggesting that cases of strong asymmetry should be short-lived and rarely observed. As a result, many examples of hybrid zones appear to be relatively stable (Barton and Hewitt 1985); however, more and more examples of asymmetric introgression are being discovered (Buggs 2007). Evidence for asymmetry can take a number of forms, including direct observation of pairing-related behaviors of hybridizing forms (Bronson et al. 2003; Leichty and Grier 2006; Charpentier et al. 2012), population genetic estimates of migration rates (Geraldes et al. 2008; Mullen et al. 2008; Nevado et al. 2011; Charpentier et al. 2012), patterns of correlation (or lack thereof) among genetic and phenotypic markers (Parsons et al. 1993; Rohwer et al. 2001; Roca et al. 2005; Wang et al. 2011; Hailer et al. 2012), and long-term observation of hybrid zone movement (Dasmahapatra et al. 2002; Carling and Zuckerberg 2011). Another, less commonly-reported pattern that suggests asymmetric hybridization is the disjunct distribution of a phenotype within the range of a phenotypically distinct form or forms with which it is hybridizing (Weisrock et al. 2005; Culumber et al. 2011).

**Figure 1:**
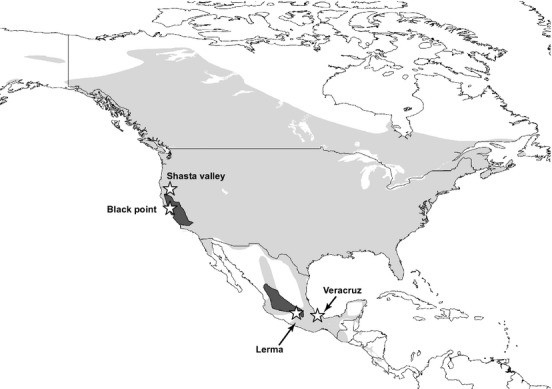
Sampling of bicolored and typical populations of the red-winged blackbird (*Agelaius phoeniceus*). The breeding range of the species as a whole is shown in light gray, and the range of bicolored phenotype blackbirds highlighted in dark gray (van Rossem 1926; F. K. Barker, unpubl. data): white stars mark the locations of genetic sampling.

Red-winged blackbirds exhibit such a mosaic pattern of phenotypic variation. Disjunct populations of blackbirds in California (the Central Valley and central coast) and central Mexico (the southern Sierra Madre Occidental and Eje Neovolcánico Transversal; see Fig. 1) share unique phenotypic characteristics that have earned them the description of “bicolored” blackbird (Mailliard 1910): (i) In males, the median coverts of the wing are black (rather than yellow as in the rest of the species' range), (ii) females are uniformly dark brown/sooty, with limited striping on the throat or upper breast (rather than buff with dark streaks). These forms are sufficiently distinct from surrounding blackbird populations that they were originally described as a separate species, *Agelaius gubernator* (reviewed in Mailliard 1910). Subsequent work has shown that both the California and Mexico populations intergrade more or less extensively with “typical” red-winged blackbirds (*Agelaius phoenicius*; Mailliard 1910; van Rossem 1926; Hardy and Dickerman 1965), and the four *A. gubernator* subspecies (*gubernator* in Mexico, and *mailliardorum*, *californicus*, and *aciculatus* in California, each differentiated by range and morphological measurements) were synonymized with *A. phoeniceus* in 1931 (Stone et al. 1931). van Rossem (1926) suggested that the bicolored forms represented an incipient species experiencing extensive hybridization with its sibling species, the typical red-winged blackbird. Here, we offer genetic evidence that disjunctly distributed but phenotypically similar populations of red-winged blackbird are each other's closest relatives, most likely representing a divergent lineage now going extinct through asymmetric hybridization.

## Materials and Methods

### Geographic sampling

Sampling was designed to include pairs of both bicolored and typical *A. phoeniceus* from geographically proximate locations in California and Mexico (Fig. 1). Importantly, distances between population pairs were short (∼400 km) compared with those within phenotypically similar populations (∼3000 km), yielding contrasting expectations of genetic diversity patterns from phenotypic similarity and isolation by distance. Our sample included a total of 78 individuals (Table S1; available on Dryad, doi: 10.5061/dryad.2d486): 20 from Black Point Sporting Club, California (38°7′59″N 122°27′9″W; referred to hereafter as “Black Point”); 18 from multiple sites in the Río Lerma valley and surroundings, Mexico (hereafter “Lerma”); 20 from Shasta Valley, California (41°42′16″N 122°28′44″W; hereafter “Shasta Valley”); and 20 from multiple sites within Veracruz, Mexico (hereafter “Veracruz”). These populations correspond to previously named subspecies *A. p. mailliardorum*, *gubernator*, *nevadensis*, and *richmondi*, respectively. Black Point and Shasta Valley samples were fresh muscle or liver tissues from birds collected in 2008 and 2009. Recent fieldwork in the upper Rio Lerma valley (pers. obs., FKB) indicates that admixture of bicolored and typical blackbirds is much more widespread than reported in the 1960s (Hardy and Dickerman 1965). In order to avoid the signal of recent admixture, Mexican samples from Lerma and Veracruz were taken from toe pads of museum specimens collected by Dickerman from 1961 to 1965. Future work will test for changes in the genetic makeup of Mexico populations since the 1960s.

### DNA extraction and mitochondrial sequencing

DNA was extracted with DNEasy kits (Qiagen, Valencia, CA) according to the manufacturer's protocols. For toe pad samples, we added 20 μmol of dithiothreitol (DTT) in aqueous solution to the digestion stage to aid in keratin proteolysis and concentrated final elutions by ethanol precipitation and resuspension of DNA in 1/5 the normal volume of buffer. We sequenced 595 bp of the mitochondrial DNA control region (CR) from each sample. The sequenced region is highly variable and information dense (Barker et al. 2011), and thus serves as an excellent marker for differentiation of recently diverged populations. For most samples, CR was amplified and sequenced in one fragment, using primers BBCR_For1 (5′-CCCCCAGTACATTTTCTTCTT-3′) and BBCR_Rev1 (5′-CCAAGTGTAGGAGGTCGGTAT-3′). Fragments were amplified by polymerase chain reaction (PCR) in 12.5 μL volumes, with 25–100 ng of template DNA, 5 pmol of each primer, 5 nmol dNTPs, 1× reaction buffer, 25 nmol MgCl_2_, and 0.3125 units of Taq polymerase (HotStarTaq; Qiagen and GoTaq; Promega, Fitchburg, WI). Cycling conditions consisted of 30 cycles of 20 s denaturation at 95°C, 20 s annealing at 55°C, and 45 s extension at 72°C, followed by a 3 min extension at 72°C. For toe pad extractions, reactions were identical to those above, but used 35 cycles instead of 30, and the time for each step was doubled (e.g., 40 s denaturation). Most PCR products from toe pads were then reamplified with 25 additional cycles prior to sequencing, in order to increase concentration of PCR product. Because we were not always able to amplify CR in one fragment from toe pad extractions, we designed the following internal primers to amplify and sequence CR in two fragments: BBCR1_intF (5′-CACCTTTGAATTCCCCTAGTCT-3′) and BBCR1_intR (5′-GCTTGGGTGYTCYTGAAGGCT-3′).

All products were purified with Exonuclease and shrimp alkaline phosphatase (USB, Cleveland, OH) and sequenced using ABI BigDye, version 3 chemistry, and an ABI 3730XL capillary sequencer (Applied Biosystems, Carlsbad, CA). All fragments were sequenced in both directions. Reads were assembled, trimmed, and edited in Sequencher, version 4.1 (Gene Codes, Ann Arbor, MI). Sequences were aligned by hand; alignment of *A. phoeniceus* CR sequences is straightforward, as insertions and deletions in this part of the CR are rare. All sequences have been deposited in GenBank (accessions KF734002–KF734078).

### Microsatellite genotyping

We genotyped each individual at five short tandem repeat (STR or microsatellite) loci (Ap49, Ap64, Ap115, Ap146, and Ap160) developed specifically for *A. phoeniceus*, which were previously tested for optimal annealing, consistent amplification, homology, and variability in both bicolored and typical populations (Barker et al. 2011). These loci were selected because they yielded the smallest expected products, a critical factor in PCR amplification from degraded material (Schneider et al. 2004). For the two California populations for which we had fresh material, we genotyped an additional five loci (Ap15, Ap38, Ap79, Ap107, and Ap144) for a total of ten. We generated fluorescently labeled fragments using nested PCR (Schuelke 2000). Each locus-specific forward primer included a 5′-M13 tail, and PCRs included this tailed primer, fluorescently labeled M13 primer, and an unlabeled reverse primer. This approach allows flexible multiplexing of fragment analysis, as any locus can be labeled with any dye. We performed reactions in 12.5 μL volume, with 25–100 ng of template DNA, 2 pmol each of the locus-specific forward and reverse primers, 0.2 pmol dye-labeled M13 primer, 5 nmol dNTPs, 1× reaction buffer, locus-specific quantity of MgCl_2_, and 0.3125 units of Taq polymerase (Qiagen HotStarTaq or Promega GoTaq). For toe pad extractions, primer quantities were adjusted to increase the quantity of fluorescently labeled PCR product. These reactions were as above, but with 1.25 pmol forward primer, 5 pmol reverse primer, and 5 pmol dye-labeled M13 primer. Cycling consisted of 25 cycles of 15 s denaturation at 95°C, 15 s annealing at primer-specific temperatures, and 20 s extension at 72°C, followed by 10 similar cycles with 53°C annealing temperature, and finally a 7 min extension at 72°C.

Fragment sizes were determined by electrophoresis on an ABI 3730XL, using the LIZ GS500 internal size standard (Applied Biosystems) and reference fragments to assess variation across runs and dyes. Size scores were estimated by the analysis of electropherograms in *GeneMarker,* version 1.91 (SoftGenetics, State College, PA). Raw size data were binned using the method of Idury and Cardon (1997) implemented by ourselves in *R* (R Development Core Team 2012). Any ambiguous calls were regenotyped or discarded, and a sample of products was sequenced to ensure locus homology. Allele bins were converted to repeat numbers by comparison with fragments with known repeat numbers determined via sequencing. All raw fragment size data, optimized bin positions, and allele designations are available in Table S2 on Dryad (doi: 10.5061/dryad.2d486).

### mtDNA genealogy

We inferred relationships among individuals from the CR sequences using Bayesian inference in *BEAST, version 1.7.2 (Heled and Drummond 2010; Drummond et al. 2012). We included sequences of two other species (10 *Agelaius tricolor* and one *Molothrus aeneus*; Barker et al. 2012) as outgroups and then performed species tree inference for the three species assuming coalescent branching within species (piecewise linear population sizes with a constant root) and a Yule process (λ∼Exp[0.1]) between. The nucleotide substitution process was modeled as HKY+G_10_ (Hasegawa et al. 1985; Yang 1994) with default priors, following previous analyses of CR in these species (Barker et al. 2012). We performed multiple unheated Markov chain Monte Carlo runs of 1·10^7^ generations and sampling every 1000, evaluated burn-in proportions on parameters using *Tracer,* version 1.5 (Rambaut and Drummond 2004), and on topologies using *AWTY* (Nylander et al. 2004). We discarded preburn-in samples and calculated nodal and parameter posteriors using *Tracer* and *TreeAnnotator* (Drummond et al. 2012).

### Summary statistics and population differentiation

We calculated summary statistics for the mitochondrial sequences using *DNAsp,* version 5.10.1, and *Arlequin,* version 3.5 (Rozas et al. 2003; Excoffier and Lischer 2010), and for STR loci using *Arlequin*. We tested for pairwise linkage disequilibrium among STR loci and for deviations from Hardy–Weinberg equilibrium in *Arlequin*. We calculated pairwise measures of population differentiation from both types of genetic data. For both the sequences and STR loci, we used *F*_ST_, which recognizes only allelic or nucleotide identity. For the STR data, we also conducted analyses using *R*_ST_ (Slatkin 1995); this metric weights difference between alleles by the square of the size difference, assuming a stepwise mutation model. Microsatellite data for these analyses included only the five loci genotyped in all populations. Finally, we summarized all measures of population differentiation by clustering populations with the unweighted paired group method using arithmetic averages (UPGMA) in PAUP* 4.0b10 (Swofford 1998).

### Individual clustering using STR data

In addition to population-level analyses, we clustered individuals using two complementary analyses of the STR data. First, we inferred genetic clusters using discriminant analysis of principal components (DAPC; Jombart et al. 2010). DAPC is a nonparametric method that does not require assumptions about allelic equilibrium. As implemented in the *R* package *adegenet,* version 1.3.4 (Jombart 2008), DAPC decomposes variation in STR alleles into principal components and uses discriminant functions constructed from those principal components to describe groupings of individuals. We identified the most likely number of clusters using *k*-means clustering, retaining principal components totaling 90% of the variation. Fit of the data to models with different numbers of clusters was assessed by the Bayesian information criterion (BIC). We then assigned individuals to clusters using discriminant functions. We determined the appropriate number of discriminant functions to retain by estimating the optimal α score, using randomization of alleles to ensure that models were not overfit. Finally, we plotted individuals on the discriminant function axes in order to visualize variation within and among the sampled populations.

Second, we conducted Bayesian clustering in *Structure,* version 2.3 (Pritchard et al. 2000), using an admixture model with correlated allele frequencies (Falush et al. 2003) but without an informative location prior (Hubisz et al. 2009). Each run included a 10,000-step burn-in, followed by 100,000 postburn-in steps. We performed 20 separate runs for each value of *k* from 1 to 6 and summarized these data using *Structure Harvester* (Earl and Vonholdt 2012). Using the median ln(*P*[*D*|*k*]) values, we estimated the posterior probability for each value of *k* and selected the value of *k* with the highest probability for subsequent analyses. We then performed 100 runs for this value of *k*, aggregated runs in *clumpp* (Jakobsson and Rosenberg 2007), and visualized results with *distruct* (Rosenberg 2004).

### Coalescent estimation of divergence with gene flow

We estimated directionality of gene flow among populations by fitting an isolation with migration model, using *IMa,* version 2.0 (Nielsen and Wakeley 2001; Hey 2010). Preliminary analyses indicated that our data were insufficient to accurately estimate parameters for models including all four populations simultaneously; therefore, we ran a number of pairwise models, with each pair of populations similar in geography or phenotype (e.g., California populations, Mexico populations, bicolored populations, typical populations.). All analyses included mitochondrial sequences and the five STR loci genotyped for all populations. Initial values of priors were estimated from the data. When run results indicated that parameter values were reaching prior bounds, upper bounds were increased and analyses rerun. For each run, we used 80 heated chains, with geometric heating and heating terms of 0.999 and 0.5. Analyses were run with indeterminate burn-in until log-probability and other parameter values appeared to stabilize, typically ∼1,000,000 generations. Following burn-in, runs were parallelized by starting five to ten separate runs from the saved MCMC state with a shorter burn-in. The number of chains and heating conditions was the same as above, and genealogies were saved every 40 generations. Postburn-in analyses were run until a total of 100,000 genealogies were saved. These separate runs were then compiled for the estimation of parameter values and model tests. We compared model fits using the Akaike's Information Criterion (AIC) and evaluated specific comparisons (e.g., nonzero migration, asymmetry of migration) with likelihood ratio tests under asymptotic expectations (Nielsen and Wakeley 2001).

## Results

### mtDNA genealogy

**Figure 2:**
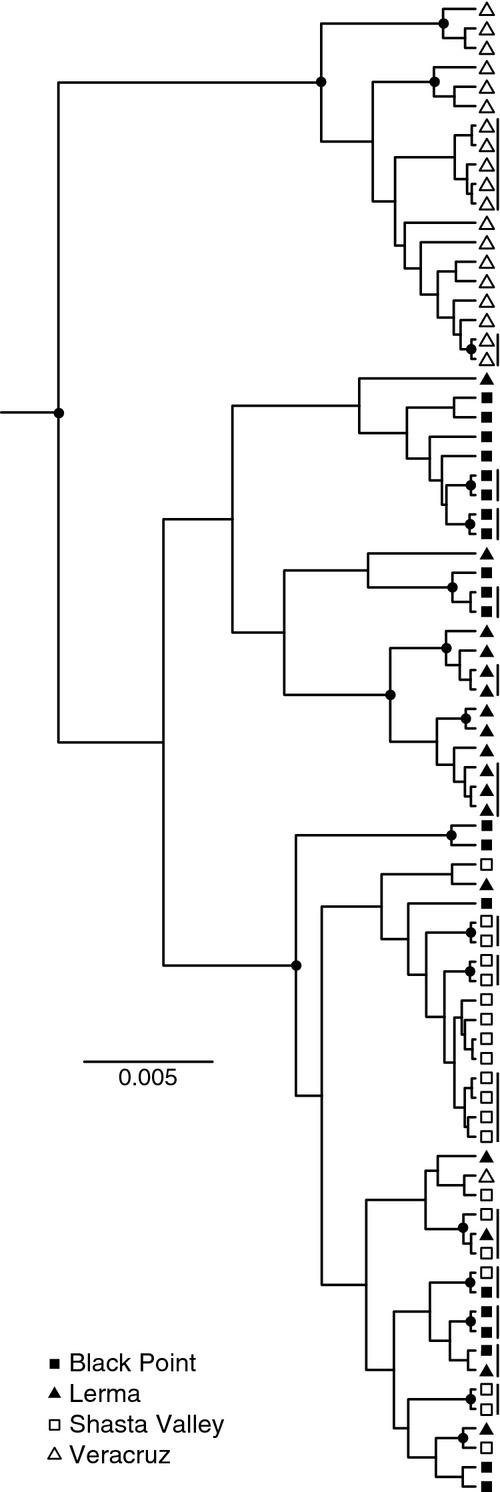
Mitochondrial genealogy of sampled *Agelaius phoeniceus* populations based on the analysis of control region sequences. Shown is the maximum clade credibility tree calculated from the posterior distribution (10,000 samples) estimated by *BEAST analysis of the *A. phoeniceus* data and two outgroups (not shown; see Materials and Methods). Nodal posterior probabilities ≥0.95 are shown by filled circles, and identical haplotypes highlighted by a line to the right of the corresponding haplotype cluster.

Haplotype diversity was high in our sampled populations (Table 1), and the few identical haplotypes were generally shared within a population (Fig. 2). In fact, we found only three cases of shared haplotypes between populations: one shared between Lerma and Black Point, another between Lerma and Shasta Valley, and a third between Black Point and Shasta Valley (Fig. 2). Although most individuals from each population cluster together, none of the four populations sampled here is monophyletic at the mitochondrial locus. Nevertheless, some general patterns are apparent. First, all but one of our samples from Veracruz formed a strongly supported monophyletic lineage most often reconstructed as sister group to all remaining blackbird mitochondrial variation. The remaining variants formed two major clusters, although only one was strongly supported (estimated posterior ≥0.95). The first (poorly supported) lineage was composed entirely of haplotypes from Black Point and Lerma, with two cases of Mexican haplotypes appearing as sister to clusters of Black Point haplotypes. The second (well-supported) lineage contained a mixture of haplotypes from all four populations, although numerically dominated by Shasta Valley. Implications of these data for population connectivity are addressed below.

**Table 1:** Mitochondrial and short tandem repeat variation in two bicolored and two typical-plumaged populations of red-winged blackbird (*Agelaius phoeniceus*)

Data	Parameter	Population			

		Black Point	Lerma	Shasta Valley	Veracruz
mtDNA	*N*	20	17	20	20
	*h*	0.979	0.971	0.900	0.937
	*S*	21	25	16	22
	η	22	26	17	22
	π	0.009	0.010	0.007	0.007
	*θ*_W_	0.010	0.013	0.008	0.010
	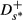	−0.404	−0.752	−0.482	−1.335
5 STR loci	*N*/locus	38.8	28.4	34.0	29.6
	# alleles/locus	8.2	8.0	8.2	6.2
	*H*_o_	0.761	0.743	0.630	0.503
	*H*_e_	0.828	0.813	0.726	0.686
	*F*_IS_	0.079	0.100	0.229	0.182
10 STR loci	*N*/locus	39.3	–	37.0	–
	# alleles/locus	8.8	–	8.9	–
	*H*_o_	0.729	–	0.725	–
	*H*_e_	0.835	–	0.789	–
	*F*_IS_	0.130	–	0.110	–

*N,* number of haplotypes sampled; *h*, haplotype diversity; *S*, number of segregating sites; η, minimum number of mutations; *p,* nucleotide diversity; *θ*_W_*,* Watterson's (1975) estimator of the neutral parameter (per site); 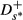, Misawa and Tajima's neutral equilibrium test (Misawa and Tajima 1997; Barker et al. 2012) with α = 0.101 (based on results from *BEAST); *N*/locus, average number of chromosomes sampled; *H*_o_, observed heterozygosity; *H*_e_, expected heterozygosity; *F*_IS_, inbreeding coefficient.

### Summary statistics and population structure

**Figure 3:**
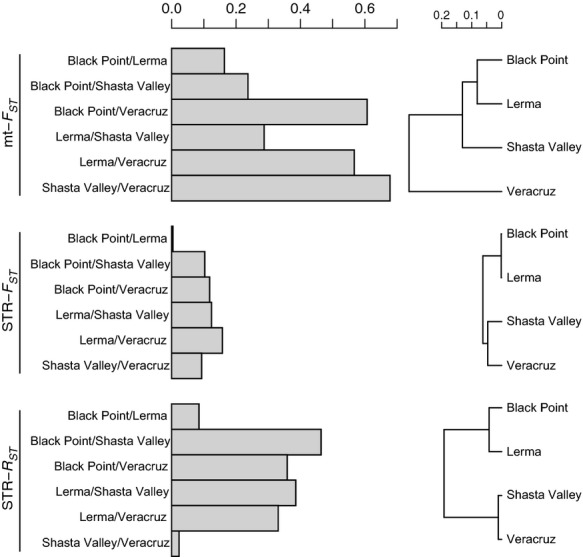
Genetic differentiation among sampled populations of *Agelaius phoeniceus*. On the left, three differentiation statistics (*F*_ST_ for mitochondrial and STR data, and *R*_ST_ for STR data only) are summarized for all pairwise comparisons of populations (all comparisons except Black Point/Lerma for STR-*F*_ST_ and Shasta Valley/Veracruz for STR-*R*_ST_ were significantly >0 by permutation test). On the right, populations are clustered by each statistic using UPGMA (Sneath and Sokal 1973).

All four populations showed substantial variation in both mitochondrial DNA and STR loci (Table 1), consistent with previous blackbird studies (Ball et al. 1988; Williams et al. 2004; Barker et al. 2012). Both mitochondrial and nuclear loci showed a wide range of genetic differentiation among populations (Fig. 3). For instance, nuclear *F*_ST_ comparisons ranged from almost zero (between Black Point and Lerma) to nearly 0.16 (between Lerma and Veracruz), and similar variability was shown by other loci and measures of differentiation (Fig. 3). Previous STR work on “typical” blackbird populations in California, Louisiana, Alberta, Manitoba, and Minnesota found much lower differentiation (*F*_ST_ = 0.002–0.023; Williams et al. 2004). Although that study used different, nonspecies-specific loci, its reported levels of polymorphism were similar, suggesting that the numbers are comparable (Jost 2008) and that the low population differentiation outside of California and Mexico (see also Gavin et al. 1991) is specific to northern and eastern populations of the species.

UPGMA phenograms constructed from the measures of population differentiation consistently united the two bicolored populations (Black Point and Lerma) to the exclusion of the two typical populations (Shasta Valley and Veracruz; Fig. 3), regardless of genetic locus. However, results from the mitochondrial sequences and STR data differ in placement of the Veracruz population. Based on the mitochondrial data, Veracruz is the most divergent population, whereas nuclear data place Veracruz with Shasta Valley. Although these two typical populations show significant differentiation with *F*_ST_, their allelic variants are apparently similar in repeat number, as *R*_ST_ values for this pair are substantially lower. Conversely, *R*_ST_ values are much higher than *F*_ST_ for the remaining comparisons, suggesting that not only do these populations differ in allele frequencies, but also in the similarity of segregating alleles.

Deviations from Hardy–Weinberg equilibrium were detected for two loci in one population each. As no population shows deviations for more than one locus, and no locus shows deviations in more than one population, these may be due to admixture or due to low-frequency null alleles. Significant linkage disequilibrium was detected among three of the STR loci in multiple populations: Ap49, Ap64, and Ap115. Linkage disequilibrium per se is expected in admixing populations and in fact provides some of the evidence for population membership (Pritchard et al. 2000). However, previous work in our laboratory suggests that these three loci may be linked to the Z chromosome (Barker et al. 2011). To ensure that our results were not biased by inclusion of all three of these loci, we reran analyses excluding two of them (Ap49 and Ap64), using either eight or three total STR loci, depending on the analysis. In all cases, measures of population differentiation and clustering of individuals with these reduced data sets did not differ qualitatively from those with full data sets, so we present results of the latter.

### Clustering of individuals using STR data

**Figure 4:**
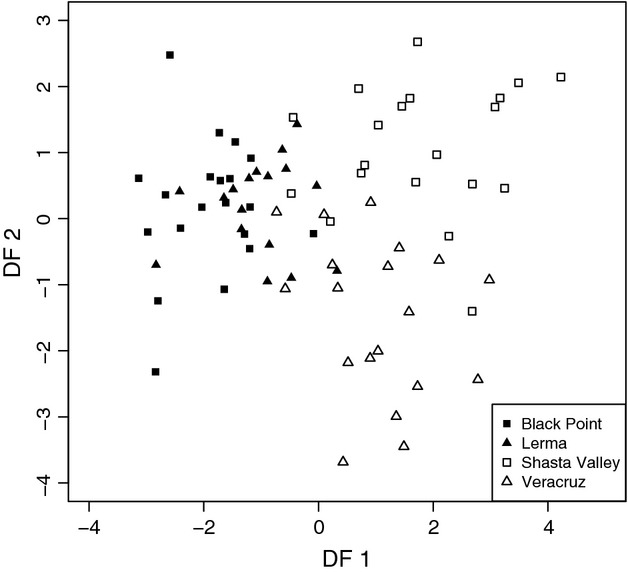
Scatterplot of individuals in the first two dimensions in a discriminant function space constructed by DAPC analysis of 10 nuclear STR loci. The optimal number of clusters in the full space was 2 (see Results).

**Figure 5:**
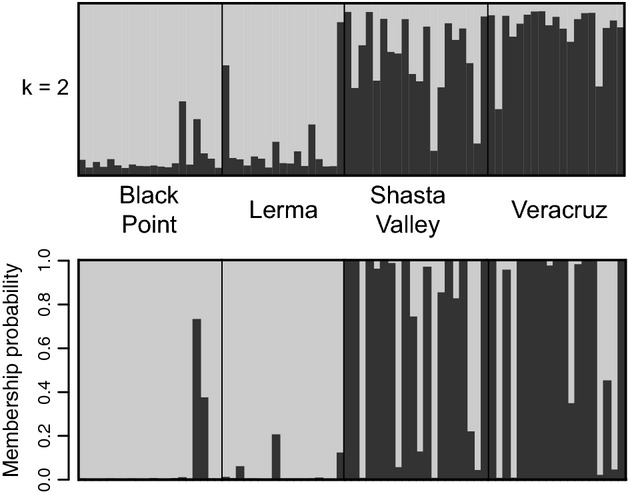
Results of individual clustering using STR data. Shown are the individual assignment probabilities to two genetic clusters (the optimal choice using both *Structure* and DAPC analysis; see Results) for both *Structure* (above) and DAPC analysis (below) of 10 nuclear loci. Each bar represents a single individual, and the proportion of shading represents the relative posterior probability of membership in each cluster. Individuals are ordered by sampled population; order of individuals is the same in each plot.

Our results from DAPC analysis were qualitatively similar with different subsets of loci, so we present results using all ten. *K*-means clustering identified two as the most likely number of genetic groups in the STR data; one cluster contained nearly all bicolored individuals and a minority of typical individuals, while a second cluster contained the remaining typical individuals (Figs. 4 and 5). In line with this result, the first discriminant function primarily differentiated bicolored and typical birds, while the second discriminant function separated the two typical populations from each other (Fig. 4).

**Figure 6:**
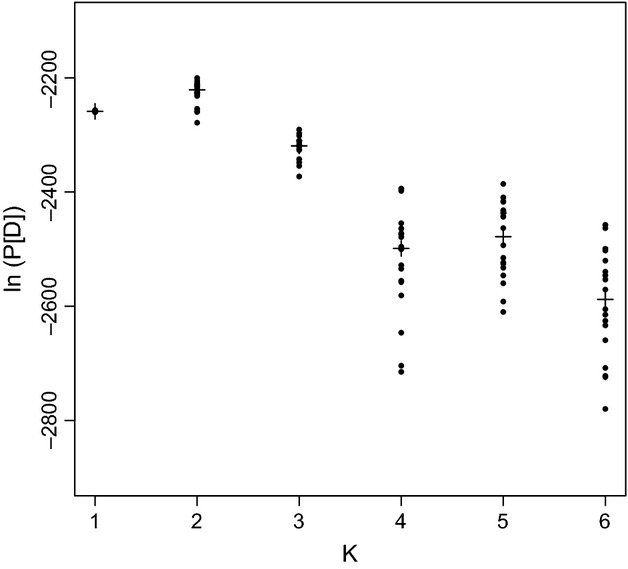
Estimated ln(*p*[Data|*k*]) for five nuclear STR loci. Shown are the estimated values for 20 separate runs at each value of *k*, using an admixture model with correlated allele frequencies and no location prior. A “+” sign marks the median values used in calculation of the posterior probability of *k*.

As with the DAPC analysis, *Structure* analyses were qualitatively similar with different sets of loci, so we report results using all ten. Bayesian analysis of these data in *Structure* consistently recovered the most appropriate value of *k* as 2 (Fig. 6), with a posterior probability ∼1.00. This result was insensitive to variations in the model and data (e.g., number of loci, independent vs. correlated allele frequencies, with and without informative location prior). In all cases, the two inferred clusters divided individuals into one group containing most or all bicolored individuals, and one group containing most or all typical individuals (Fig. 5). In runs assuming *k* = 3, one cluster containing bicolored birds was still recovered, while the other two clusters varied in their composition, sometimes corresponding to the two typical populations, and sometimes containing ∼50% of each individual from both typical populations (results not shown). The concordance between the DAPC and *Structure* results is notable, as it indicates that the inferred number of genetic clusters is insensitive to the different assumptions of these two methods. In addition, the assignment of individuals to clusters was strongly correlated between the two methods (Fig. 5; Pearson's *r*^2^ of logit-transformed assignment probabilities = 0.79).

### Coalescent estimation of divergence with gene flow

We found strong evidence of gene flow between populations. First, in all cases, the model with minimum AIC included migration (Table 2). In addition, with the exception of migration from Lerma to Black Point, modal migration rates were >0, and 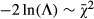 for comparisons of no migration to symmetrical migration ranged from 22 (Black Point/Shasta Valley) to 1586 (Black Point/Lerma), with 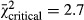 at α = 0.05. In addition, we found evidence that migration rates among populations were not uniform. In two of the four comparisons, the model with minimum AIC was one in which one of the two migration rates was constrained to zero (Table 2). The first of these comparisons, between Black Point and Lerma, also showed significant asymmetry in comparisons between constrained and unconstrained dispersal models (−2ln[Λ] = 6.8, *P* < 0.01). This model implies dispersal from California to Mexico, with little or no dispersal in the other direction (Table 3). The second comparison, between Black Point and Shasta Valley, showed only weak support for an unconstrained model of dispersal (−2ln[Λ] = 1.1, *P* = 0.15), in this case implying higher dispersal rates from Shasta Valley to Black Point than the reverse.

**Table 2:** Model comparisons from coalescent analysis of blackbird mitochondrial and microsatellite data under an isolation with migration model

Model constraints	K	Populations compared (1/2)			

		Black point/Lerma	Black point/Shasta Valley	Lerma/Veracruz	Shasta Valley/Veracruz
Full model	13	42.4	40.8	47.0	48.1
*θ*_1_ = *θ*_2_	12	41.1	39.8	48.2	46.4
*m*_1→2_ = *m*_2→1_	12	47.2	39.9	**45.0**	47.7
*m*_1→2_ = *m*_2→1_ = 0	11	1630.8	622.2	258.6	617.2
*m*_1→2_ = 0	12	1561.8	**38.2**	71.4	53.3
*m*_2→1_ = 0	12	**40.4**	38.6	49.8	47.5
*θ*_1_ = *θ*_2_ and *m*_1→2_ = *m*_2→1_	11	45.4	39.7	47.2	**46.2**

*θ*_i_ = population size, *m*_*i*→*j*_ = (forward) migration rate between populations *i* and *j*.

Shown are the number of estimated parameters and Akaike's Information Criterion values for the seven models compared in *IMa,* version 2.0, with the best model for each population pair under that criterion highlighted in bold.

**Table 3:** Parameter estimates from coalescent analysis of blackbird mitochondrial and microsatellite data under an unconstrained isolation with migration model. Modal parameter values (descendant and ancestral population parameters *θ*_*i*_, and forward-time migration rates *m*_*i*→*j*_) and approximate 95% highest posterior density intervals are shown for pairwise analyses of the sampled populations

Population 1	Population 2	*θ*_1_	*θ*_2_	*θ*_A_	*t*_0_	*m*_1→2_	*m*_2→1_
Black Point	Lerma	16.43 (7.65, 26.65)	12.97 (4.55, 28.85)	77.52 (0.00, 877.5)	2.44 (0.28, 79.96^1^)	1.27^2^ (0.48, 3.69)	0.01^2^ (0.00^1^, 1.08)
Black Point	Shasta Valley	10.86 (4.52, 19.78)	7.02 (2.90, 13.70)	80.24 (13.50, 403.5)	1.49 (0.46, 16.93)	0.26 (0.00, 1.29)	0.32 (0.00, 1.74)
Lerma	Veracruz	16.11 (9.13, 26.73)	8.21 (4.53, 13.97)	5781 (1925, 45,525^1^)	18.60 (6.84, 57.16)	0.08 (0.01, 0.33)	0.09 (0.01, 0.32)
Shasta Valley	Veracruz	11.21 (6.02, 19.02)	9.00 (4.73, 15.38)	3630 (825, 39,725^1^)	10.92 (4.92, 30.12)	0.12 (0.01, 0.67)	0.25 (0.02, 0.85)

1 Posterior distribution arbitrarily truncated due to prior limits (maxima on *N*_A_ and *t*_0_).

2 *m* values significantly (*P* < 0.05) asymmetric by likelihood ratio test under asymptotic assumptions.

## Discussion

### Past genetic connections between disjunct populations of bicolored blackbird

In this study, we have sampled mtDNA and STR variation from phenotypically pure populations of bicolored and typical blackbirds in both California and Mexico. Ongoing work is addressing genetic and phenotypic variation at a finer scale, but we expect analyses reported here that exclude hybridizing populations best represent patterns of relatedness between these forms. These data demonstrate that our samples of bicolored blackbirds form a genetically cohesive unit differentiated from typical blackbirds, despite the fact that these populations are separated by over 3000 km. Within this context, several results are of particular note. First, bicolored blackbirds do not comprise a single mitochondrial lineage that is reciprocally monophyletic from typical blackbirds, nor are there two major mitochondrial lineages with significant frequency differences between these two phenotypes (Fig. 2). Thus, if these forms originated by isolation in allopatry, it is unlikely that the isolation was of long duration or that either of the isolated populations experienced significant genetic bottlenecks (Rosenberg 2007). Despite the lack of reciprocal monophyly, estimates of population differentiation based on mitochondrial sequences (Fig. 3), which have a direct relationship to gene flow between populations (Hudson et al. 1992), indicated a closer relationship between the two bicolored populations than between any other pair. Likewise, two measures of population differentiation at nuclear loci indicate a close genetic relationship between the bicolored populations, and both nonparametric and parametric clustering analyses of the same data identified two genetic clusters largely corresponding to the phenotype. Finally, mean migration rate estimates ([*m*_1→2_ + *m*_2→1_]/2) between Black Point and Lerma estimated by coalescent analyses of all the data were higher than between any other pair, although this difference is not statistically significant (Table 3). Based on these data, we suggest that the current mosaic phenotypic distribution of *A. phoeniceus* is consistent with a scenario of “incomplete speciation,” where two forms differentiated in allopatry have come into broad secondary contact.

The genetically close relationship between bicolored *A. phoeniceus* populations, despite their disjunct distribution, suggests either that they were once continuously distributed between California and Mexico or that one population founded the other by long-distance dispersal. If the latter is true, estimated migration rates from our coalescent analyses are consistent with founding of the Mexico population from California (Table 1). However, neither the within-population measures of diversity (Table 1) nor the mtDNA genealogy (Fig. 2) show expected signatures of a founding event. Based on this evidence, and given that allopatric differentiation followed by secondary contact appears to be the norm for most organisms examined to date (e.g., Harrison 1993; Arnold 2006), we favor the former scenario. This implies that the typical form of blackbird has expanded and is impinging on bicolored populations, contributing to their current restricted distributions. Consistent with this idea, our data favor a model of asymmetric migration from typical to bicolored populations in California (Table 2). A similar result was not obtained for Mexican populations, but migration estimates to and from Veracruz were among the lowest we observed (Table 3), especially for the mitochondrial data, which show near reciprocal monophyly between the Mexican populations (Fig. 2). Sampling of other lowland Mexican populations of typical redwings (e.g., in Morelos or Puebla) may prove more informative in this regard. If the disjunct distribution of the bicolored phenotype is due to asymmetric hybridization, then this distinctive form will most likely be lost to extinction unless this process is counteracted by local adaption or changes in migration rates (Rhymer and Simberloff 1996).

### Mosaic phenotypic distributions as evidence of asymmetric hybridization

The unique combination of evolutionary events in any species' history can generate a near-infinite variety of patterns of intraspecific phenotypic variation. However, numerous generalities can be found among these diverse patterns. Spatially, variation can occur over very broad geographic scales (e.g., wide clines), to abrupt transitions (e.g., narrow or step clines). The presence of multiple abrupt transitions between phenotypes yields a “mosaic” pattern, where isolated populations may all be different from one another, or where some populations (adjacent or otherwise) share phenotypic characteristics. Mosaic patterns of variation, with geographically disjunct but phenotypically similar forms – as seen in the red-winged blackbird – are particularly interesting, as there are relatively few evolutionary processes that can produce them, and these processes are of intrinsic interest. On the one hand, adaptation in a heterogeneous landscape can drive population differentiation, and similar phenotypes may arise if disjunct regions share similar selective environments (parallel evolution). Alternatively, differentiation in allopatry, followed by partial displacement of one postisolation form by another, can yield similar disjunct patterns. Examples of both these patterns have been reported previously, but are relatively rare.

Previous studies have identified several cases of disjunctly distributed forms evolving convergently or in parallel due to similar selective regimes. Extensive research on variation in the three-spined stickleback (*Gasterosteus aculeatus*) has revealed repeated, independent evolution of multiple locally adapted freshwater forms from an oceanic ancestor. These disjunct forms show consistent phenotypic changes related to locomotion and feeding in different habitats (Schluter and McPhail 1992; Berner et al. 2009; Kaeuffer et al. 2012), and genetic data show these forms clustering by lake basin, rather than by phenotype (Thompson et al. 1997; Taylor and McPhail 1999, 2000; Hendry and Taylor 2004; Berner et al. 2009). Additional research has shown that similar phenotypes may have different genetic underpinnings (Colosimo et al. 2005; Chan et al. 2010), further supporting parallel evolution as the explanation for their disjunct distribution. Other notable cases of mosaic distribution due to selection involve evolution of coat color in mammals. The rock pocket mouse (*Chaetodipus intermedius*) has evolved dark coat coloration on multiple disjunct lava flows in the desert southwest. Although quite similar in pelage, these populations are not distinct from one another or surrounding populations based on mtDNA (Nachman et al. 2003; Hoekstra et al. 2004), and it appears that the genetic basis of coat color differs among populations (Hoekstra and Nachman 2003). A similar explanation has been found for mosaic coat color distributions in beach mice, with apparently independent origins of a similar locally adapted phenotype (*Peromyscus polionotus*; Steiner et al. 2009).

By contrast, mosaic patterns have also been observed that appear to have historical rather than selective origins. Notably, secondary contact and hybridization between differentiated but closely related forms can yield patterns similar to parallel local adaptation. For example, the high-elevation salamander *Plethodon shermani* is disjunctly distributed on mountain ranges in the southern Appalachians, but each population hybridizes freely with the closely related intermediate-elevation species *Plethodon teyahalee* (Hairston et al. 1992; Weisrock et al. 2005). This yields a mosaic of high-elevation unspotted red-legged forms embedded in a matrix of intermediate-elevation spotted dark-legged forms. The genetic uniformity of *P. shermani* across disjunct mountain ranges (as measured by allozyme data) suggests that this pattern is an historical species difference rather than local adaptation, most likely due to contraction of *P. shermani* and expansion of *P. teyahalee* distributions since the last deglaciation (Weisrock et al. 2005). Similar cases of closely related hybridizing forms can be found in fish distributed in river systems, where isolated, disjunct headwater populations form multiple hybrid zones with a widespread, downstream population (Nolte et al. 2009; Culumber et al. 2011).

We argue that the disjunct distribution of bicolored blackbirds offers another example of historically rather than selectively determined mosaic distribution. Until this study, there was no evidence that Californian and Mexican populations of bicolored blackbirds were united to the exclusion of typical forms, as should be expected if their disjunct distribution were due to range dynamics (e.g., expansion of typical red wings at the expense of bicolors) rather than local selection. Notably, there is nothing apparent in the environments of central Mexico and California that might select for the observed plumage differences, particularly parallel changes in both male and female plumage. We are currently conducting analyses of environmental similarities between these regions using niche modeling (Peterson et al. 2011) to more conclusively evaluate their potential role. It has been suggested that bicolored plumage may have evolved in response to interspecific competition with tricolored blackbirds (*A. tricolor*), which are behaviorally dominant to red wings and currently sympatric with them in California (Orians and Collier 1963); however, tricolored blackbirds do not currently occur in central Mexico. Taken together, evidence for genetic relatedness of bicolored populations and lack of a convincing source of selection driving convergence favor an historical explanation of bicolored blackbird distribution. Importantly, this does not preclude a role for the selection in this process: The asymmetric pattern of range expansion may have its roots in selected differences between these two interacting forms. This possibility could be evaluated by characterizing the zones of intergradation where the two forms meet: in the absence of selection favoring one or the other form, we would expect broader, more diffuse zones of contact.

One potential mechanism for asymmetric hybridization lies in plumage and song differences between male bicolored and typical *A. phoeniceus*. Experiments with typical *A. phoeniceus* have demonstrated the importance of the male's contrasting wing coverts and song for mate attraction and territory maintenance (Peek 1972; Roskaft and Rohwer 1987; Searcy and Yasukawa 1990; Smith 1972; Yasukawa et al. 1980, 2009; but see Westneat 2006; Yasukawa et al. 2010). Plumage clearly differs between bicolored and typical red wings: work on song differentiation in these forms is ongoing. Experimental work on the bicolored phenotype would help determine whether these trait differences correspond to the behavioral dominance by typical birds, which could explain asymmetry in hybridization through differential reproductive success.

### Asymmetric hybridization and speciation

Cases of asymmetric hybridization between closely related but incompletely isolated forms offer insight into the processes influencing species differentiation at its earliest stages. Recognition and analysis of such cases contributes significantly to the study of speciation. In particular, the fact of asymmetric range expansion reveals the existence of biologically meaningful differentiation – be it demographic, behavioral, or otherwise – between hybridizing forms. Comparative analysis of multiple lineages showing such characteristics may reveal generalities regarding the selective factors that drive such differences (e.g., environmental characteristics in a given refugial area) or the lack of such (e.g., random drift of female preference). Here, we offer red-winged blackbirds (*A. phoeniceus*) as one of a growing list of examples of asymmetric hybridization and suggest that the biological determinants of this phenomenon in blackbirds merit additional study.

## Data Accessibility

Data deposition: sequences to GenBank, sample and STR data to Dryad.

## Supporting Information

**Table S1.** Specimens of *Agelaius phoeniceus* included in this study.

**Table S2.** Short tandem repeat data for four populations of *Agelaius phoeniceus*.
